# Immuno-PET imaging for non-invasive assessment of cetuximab accumulation in non-small cell lung cancer

**DOI:** 10.1186/s12885-019-6238-4

**Published:** 2019-10-24

**Authors:** Aiko Yamaguchi, Arifudin Achmad, Hirofumi Hanaoka, Yusri Dwi Heryanto, Anu Bhattarai, Ratianto, Erdene Khongorzul, Rini Shintawati, A. Adhipatria P. Kartamihardja, Ayaka Kanai, Yumi Sugo, Noriko S. Ishioka, Tetsuya Higuchi, Yoshito Tsushima

**Affiliations:** 1 Department of Bioimaging Information Analysis, Gunma University Graduate School of Medicine, 3-39-22 Showa-machi, Maebashi, 371-8511 Japan; 2 Present address: Texas Therapeutics Institute, The Brown Foundation Institute of Molecular Medicine, The University of Texas Health Science Center at Houston, 1881 East Road, Houston, TX 77054 USA; 3 Department of Diagnostic Radiology and Nuclear Medicine, Gunma University Graduate School of Medicine, 3-39-22 Showa-machi, Maebashi, 371-8511 Japan; 4 Present address: Department of Nuclear Medicine and Molecular Imaging, Faculty of Medicine, Universitas Padjadjaran, Bandung, West Java 40161 Indonesia; 5 Oncology and Stem Cell Working Group, Faculty of Medicine, Universitas Padjadjaran, Bandung, West Java 40161 Indonesia; 6 Project “Medical Radioisotope Application”, Department of Radiation-Applied Biology Research, Takasaki Advanced Radiation Research Institute, Quantum Beam Advanced Research Directorate, National Institutes for Quantum and Radiological Science and Technology, 1233 Watanuki, Takasaki, 370-1292 Japan

**Keywords:** Immuno-PET, Non-small cell lung cancer, Cetuximab, ^64^Cu, Personalized medicine, EGFR

## Abstract

**Backgrounds:**

Overexpression of epidermal growth factor receptor (EGFR) has been established as a valid therapeutic target of non-small cell lung cancer (NSCLC). However, the clinical benefit of cetuximab as an EGFR-targeting drug is still controversial, partially due to the lack of effective means to identify suitable patients. This study aimed to investigate the potential of radiolabeled cetuximab as a non-invasive tool to predict cetuximab accumulation in NSCLC tumor xenografts with varying EGFR expression levels.

**Methods:**

The NSCLC tumors in model mice were subjected to in vivo biodistribution study and positron emission tomography (PET) imaging 48 h after injection of either ^111^In- or ^64^Cu-labeled cetuximab. The EGFR expression levels of NSCLC tumors were determined by ex vivo immunoblotting.

**Results:**

We found that tumors with high EGFR expression had significantly higher [^111^In]In-DOTA-cetuximab accumulation than tumors with moderate to low EGFR expression (*P* < 0.05). Strong correlations were found between [^111^In]In-DOTA-cetuximab tumor uptake and EGFR expression level (*r* = 0.893), and between [^64^Cu]Cu-DOTA-cetuximab tumor uptake with EGFR expression level *(*r = 0.915). PET imaging with [^64^Cu]Cu-DOTA-cetuximab allowed clear visualization of tumors.

**Conclusion:**

Our findings suggest that this immuno-PET imaging can be clinically translated as a tool to predict cetuximab accumulation in NSCLC cancer patients prior to cetuximab therapy.

## Background

Non-small cell lung cancer (NSCLC) remains a deadly cancer worldwide, even with advances in treatment strategies such as molecular targeted therapy and immunotherapy [1]. Overexpression of epidermal growth factor receptor (EGFR) plays a role in NSCLC, making anti-EGFR drugs an attractive therapeutic option for this cancer. Tyrosine kinase inhibitors (TKIs) targeting EGFR are currently recommended as first-line therapy in patients with advanced NSCLC harboring an EGFR tyrosine-kinase domain mutation. However, acquired resistance to TKIs is common and their modest effect in NSCLC patients without EGFR mutation necessitates alternative therapeutic approaches targeting EGFR [2].

Cetuximab, a recombinant, human/mouse chimeric monoclonal antibody that specifically targets the extracellular domain of EGFR, has demonstrated favorable efficacy in combination with platinum-based chemotherapies, but identification of patients likely to benefit from these therapies remains challenging [3–5]. Studies suggest that strong overexpression of EGFR rather than other factors including KRAS mutation status is a determinant factor for the treatment efficacy of cetuximab in NSCLC patients. However, it is still unclear whether positivity in immunohistochemistry (IHC) or Fluorescent in situ Hybridisation (FISH) score and/or squamous histology can be reliably predictive, presumably due to the heterogeneity of EGFR expression within tumors and/or limitations related to biopsy-based assessment such as limited tissue sampling [6, 7]. Another approach that could assess EGFR status within the entire tumor throughout the body could potentially provide more comprehensive information to predict whether a patient will respond to cetuximab treatment.

Molecular imaging with radiolabeled antibodies, including immuno-positron emission tomography (PET) imaging, can provide quantitative information about antibody uptake at a whole-body level in a non-invasive fashion [8]. Immuno-PET has shown potential for the assessment of biomarker expression status and/or prediction of clinical response [9, 10]. Studies found a significant association between the tumor uptake of copper-64 (^64^Cu) labeled cetuximab ([^64^Cu]Cu-DOTA-cetuximab) and the expression levels of EGFR protein in cervical cancer cell lines [11] and in xenograft mouse models with various cancer types [12, 13]. By contrast, some studies have found disparity between the expression levels of EGFR and tumor uptake of radiolabeled cetuximab in several tumor xenograft models from different origins, implying the influence of other factors such as pharmacokinetics and dynamics for cetuximab accumulation in tumors [14, 15].

Considering the disease heterogeneity of NSCLC, the applicability of [^64^Cu]Cu-DOTA-cetuximab for non-invasive assessment of EGFR expression status in NSCLC warrants further validation in pre-clinical models. In this study, we evaluated the usefulness of [^64^Cu]Cu-DOTA-cetuximab for the selection of EGFR-overexpressing NSCLC tumors using xenograft mouse models with human NSCLC cell lines having various EGFR protein expression levels.

## Methods

Cetuximab was kindly provided by Merck KgaA (Darmstadt, Germany). The bifunctional chelating agent p-SCN-Bn-DOTA, or 2-(4-isothiocyanatobenzyl)-1,4,7,10-tetraazacyclododecane-1,4,7,10-tetraacetic acid, was purchased from Macrocyclics (Dallas, TX, USA). Copper-64 (150–300 MBq) was produced on a biomedical cyclotron CYPRIS HM-18 (Sumitomo Heavy Industries Ltd., Tokyo, Japan) at Gunma University Hospital. Indium-111, in form of InCl_3_ (74 MBq/mL) was obtained from Nihon Medi-Physics (Tokyo, Japan).

### Cell lines and xenografts

The animal studies were performed in accordance with our institutional guidelines and were approved by the Local Animal Care Committee of Gunma University (approval number: 17–035). Human NSCLC cell lines H358 (bronchioalveolar carcinoma, ATCC CRL-5807), H441 (papillary adenocarcinoma, ATCC HTB-174), H460 (large cell lung cancer, ATCC HTB-177), H520 (squamous cell carcinoma, ATCC HTB-182), H1299 (carcinoma, ATCC CRL-5803), H1650 (adenocarcinoma; bronchoalveolar carcinoma, ATCC CRL-5883), and HCC827 (adenocarcinoma, ATCC CRL-2868) were obtained from ATCC (Manassas, VA, USA), and EBC1 (squamous cell lung carcinoma, JCRB0820) was obtained from Japanese Collection of Research Bioresources (Tokyo, Japan). All cell lines were grown monolayers in RPMI 1640 medium (Wako, Osaka, Japan) supplemented with 10% heat-inactivated FBS (Nichirei Bioscience, Tokyo, Japan) and 1% antibiotic (0.1 mg/mL penicillin and 100 U/mL streptomycin, Wako). The EGFR-null H520 cell line was used as a negative control to assess non-specific tumor uptake of radiotracer. All cell lines were cultured in a humidified atmosphere comprising 5% CO_2_ and 95% air at 37 °C. Five-weeks-old female athymic Balb/c nude mice (17–20 g) were purchased from Japan CLEA (Tokyo, Japan) or Japan SLC (Shizuoka, Japan) and allowed to acclimatize for one week in the animal facility before any intervention was initiated. Mice were socially housed (4–5 animals per cage) in cages in an air-conditioned room at 28 °C under a 12 h light/dark cycle with access to food and tap water ad libitum during all the experiment. Lung tumor xenografts were prepared by subcutaneous injection of 5 × 10^6^ cells in 100 μL PBS suspension in the dorsal flank of the mice in awake. Mice were randomly assigned to each experiment when the tumor size become approximately 100–300 mm^3^ (2–4 week after the tumor implantation).

### Westernblot analysis for EGFR expression

Xenografts mice (*n* > 2 for each tumor) were euthanized by cervical dislocation and tumors were collected. The western blot analysis was performed according to the procedure previously described [12]. Anti-EGFR (#2232; Cell Signaling, Beverly, MA, USA) or anti β-actin (clone AC-15; Sigma-Aldrich, Saint Louis, MO, USA) was used as primary antibody. Membranes were visualized by scanning using the ImageQuant™ LAS 4010 imager (GE Healthcare, Piscataway, NJ, USA) and the obtained bands were densimetry analyzed using ImageJ 1.47 software. The data were normalized over EGFR-null control H520. Based on the adjusted band density, the EGFR expression levels were classified in three categories; > 20: highly-overexpressing, 10 to 20: high, 1 to 10: low to moderate.

### DOTA conjugation

Cetuximab (2 mg/mL) was buffer-exchanged into borate-buffered saline (0.1 M, pH 8.5) in Vivaspin (Sartorius Stedim Biotech, Aubagne, France). To the concentrated cetuximab was added p-SCN-Bn-DOTA dissolved in N,N-dimethylformamide (10:1 to cetuximab). The resulting mixture was incubated overnight at 37 °C, and then unconjugated DOTA was removed by using size-exclusion column (Bio-spin 6 Tris column, Bio-Rad Laboratories, Hercules, CA, USA) and ultrafiltration (Vivaspin). The protein concentration of the resulting DOTA-cetuximab was determined by using a Nanodrop spectrophotometer (Thermo Scientific, Wilmington, DE, USA).

The immunoreactivity of DOTA-cetuximab was evaluated in a NSCLC cell line (H460) according to a method described previously [16]. No significant effect of DOTA-conjugation was observed.

### Preparation of ^64^Cu or ^111^In-labled DOTA-cetuximab

For ^64^Cu labeling, DOTA-cetuximab (500 μg) was dissolved in sodium acetate buffer (0.25 M, pH 5.5) and then added to the dried ^64^CuCl_2_ (150–300 MBq). The resulting mixture was incubated for 1 h at 40 °C. An aqueous solution of EDTA (100 mM, 5 μL) was added to quench the unconjugated ^64^Cu. Purification of [^64^Cu]Cu-DOTA-cetuximab was carried out using PD-10 desalting column (GE Healthcare). [^111^In]In-DOTA-cetuximab was obtained by a similar procedure. The radiochemical yield and radiochemical purity were determined using an instant TLC developed with saline. After purification, the radiochemical purities of both [^64^Cu]Cu-DOTA-cetuximab and [^111^In]In-DOTA-cetuximab were more than 99%. The specific activity of the final product per milligram cetuximab was 2–3 MBq for ^111^In. Due to the varying radiochemical yields, the specific activity of [^64^Cu]Cu-DOTA-cetuximab ranged 30–200 MBq/mg.

### Biodistribution study

To detect and examine in vivo behavior of radiolabeled-cetuximab in xenografts with various lung cancer cell lines, [^111^In]In-DOTA-cetuximab (protein dose: 20 μg) were intravenously injected with 30 kBq via the tail vein in awake mice (*n* ≥ 5 for each group). Mice were euthanized by decapitation at 48 h after injection. Tissues of interest were collected and weighed, and the radioactivity was measured using an automated γ-counter ARC-7001 (Hitachi Aloka Medical, Tokyo, Japan). The radiotracer uptake was expressed as percentage of injected dose/g of tissue (%ID/g).

### PET imaging

To study PET usefulness for the assessment of EGFR expression level in vivo, xenografts of lung cancer cell lines (*n* = 2) were intravenously injected with 2–20 MBq [^64^Cu]Cu-DOTA-cetuximab via the tail vein in awake. To minimize the influence of specific activity, protein dose was fixed to 100 μg by the addition of non-radiolabeled cetuximab. Considering the pharmacokinetics of monoclonal antibody [8] and the physical half-life of ^64^Cu (12.7 h), PET images were taken 48 h after injection. After anesthetization by isoflurane inhalation (2.5% in an air mixture), mice were imaged with a small-animal PET scanner (Transaxial FOV: 10 cm, axial FOV: 12.7 cm, resolution at the center of FOV: 1.4 mm, Inveon, Siemens, Knoxville, TN, USA). To obtain images with equivalent qualities, the acquisition time was adjusted based on the activity dose. The acquisition time was 20 min, 60 min, or 120 min for 20 MBq, 4 MBq, or 2–3 MBq activity dose, respectively. In all studies, PET scanning was performed with animals over a heating pad heated at 37 °C. After the PET scan, mice were euthanized by cervical dislocation. The energy window was set between 350 and 650 keV. The imaging data were reconstructed using a 3-D ordered-subsets expectation maximization algorithm. Attenuation correction and scattering correction were not applied. All images were quantified for tumor radiotracer uptake using the Inveon Research Workplace workstation (Siemens). Uptake of [^64^Cu]Cu-DOTA-cetuximab in the tumor was expressed as average of standardized uptake values (SUVmean). The SUV was determined by using the following equation: SUV = activity in a ROI (MBq/cc)/[injected dose (MBq)/body weight (g)]. Region of interests (ROI) were manually drawn to contour the tumors three times each by two investigators (HH, 10 years of experience, AK2, 1 year of experience). Tumor outlines were defined by the pixel containing SUV higher than 0.6. There was no noticeable difference in intra- and interobserver variability.

### Statistical analysis

The GraphPad Prism software (GraphPad Software, La Jolla, CA, USA) was used for statistical analysis. Data are expressed as means ± SDs where appropriate. In the biodistibution study with [^111^In]In-DOTA-cetuximab, comparison of means was performed using one-way ANOVA followed by Tukey’s test. For the comparison between tumor [^111^In]In-DOTA-cetuximab uptake, [^64^Cu]Cu-DOTA-cetuximab uptake and adjusted EGFR band density, simple correlations between variables were analyzed using Pearson’s correlation coefficient. *P*-values < 0.05 were considered statistically significant.

## Results

### EGFR expression in NSCLC cell lines

**Fig. 1:**
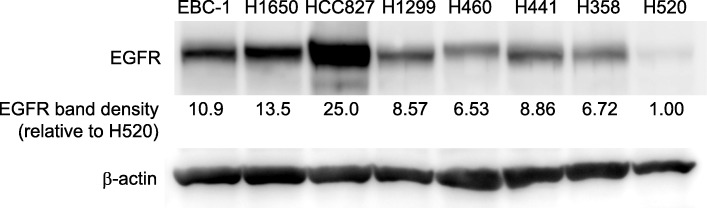
Epidermal growth factor receptor (EGFR) expression in eight non-small cell lung cancer (NSCLC) cell lines. Densitometric intensities of EGFR are presented as folds relative to H520 band density (1.0). β-actin was used as a loading control

Immunoblot analysis of the xenograft tumors showed that the eight NSCLC cell lines express various levels of EGFR (Fig. 1). Semi-quantitative confirmation of EGFR expression showed that HCC827 surpasses the rest (EGFR band density of 25.0, relative to EGFR-null H520), followed by H1650 and EBC-1 (13.5 and 10.9, respectively). Other EGFR-positive cell lines showed modest expression levels ranging from 6.53 to 8.86.

### Biodistribution of [^111^In]in-DOTA-cetuximab

**Fig. 2:**
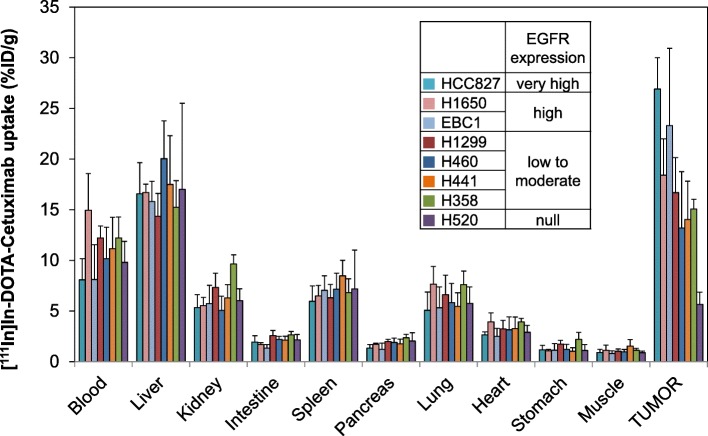
Biodistribution of [^111^In]In-DOTA-cetuximab at 48 h in mice xenograft models with non-small cell lung cancer (NSCLC) tumors with varying epidermal growth factor receptor (EGFR) expression levels. Each data point represents the mean ± SD of *n* = 5 to 7 per tumor model

Figure 2 summarizes [^111^In]In-DOTA-cetuximab uptakes in major organs and tumors 48 h after injection in NSCLC tumor xenografts (*n* = 5 to 7/group). The tumor uptake levels of [^111^In]In-DOTA-cetuximab in EGFR-positive xenografts were significantly higher than that of EGFR-null H520 tumors (*P* < 0.05). The uptake level of [^111^In]In-DOTA-cetuximab in the EGFR highly-overexpressed HCC827 tumor (*n* = 7) was significantly higher than those in other cell lines except for EBC-1 (26.9 ± 3.10%ID/g, *P* < 0.05). Although large variation was observed, EBC-1 xenografts (*n* = 5), which had relatively high EGFR expression levels, showed [^111^In]In-DOTA-cetuximab uptake levels significantly higher than xenografts with low to moderate EGFR expression levels (H358, H441, H460) (23.3 ± 7.63%ID/g, *P* < 0.05). H1650 also showed a relatively high uptake level of [^111^In]In-DOTA-cetuximab (18.4 ± 3.59%ID/g, n = 5) although the difference was not statistically significant compared to the cell lines with moderate to low EGFR expression levels (H358, H441, H460, and H1299). The other tumors derived from 4 NSCLC cell lines with moderate to low EGFR expression levels showed comparable uptake levels of [^111^In]In-DOTA-cetuximab. The radioactivity uptakes of H358 (*n* = 6), H441 (n = 6), H460 (n = 6), and H1299 (n = 6) tumors were 15.1 ± 0.96%ID/g, 14.0 ± 3.77%ID/g, 13.2 ± 5.56%ID/g, and 16.7 ± 3.46%ID/g, respectively. Accumulation in normal organs was similar in all groups.

### PET imaging of [^64^Cu]cu-DOTA-cetuximab

**Fig. 3:**
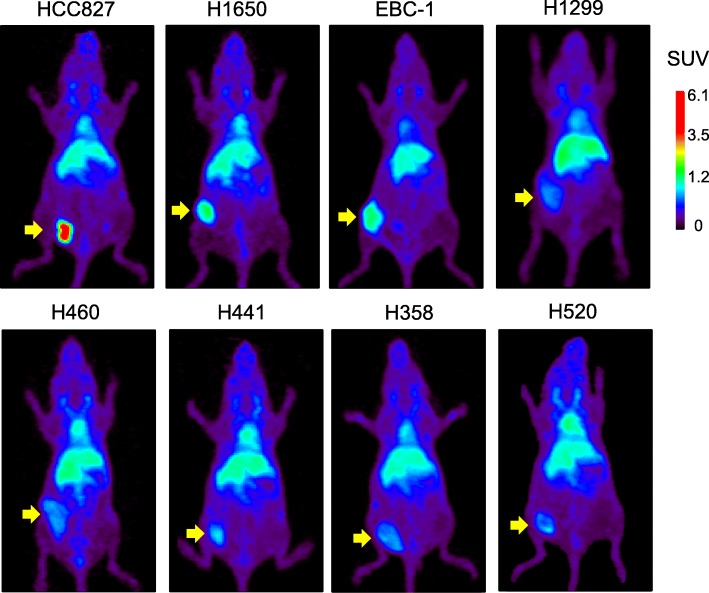
Representative [^64^Cu]Cu-DOTA-cetuximab positron emission tomography (PET) images in mice xenograft models with non-small cell lung cancer (NSCLC) tumors with varying epidermal growth factor receptor (EGFR) expression levels at 48 h. Arrows indicate the location of tumors

The whole-body distribution and tumor-targeting efficiency of cetuximab was visualized non-invasively using small-animal PET imaging at 48 h after injection of [^64^Cu]Cu-DOTA-cetuximab in mice with NSCLC xenografts (*n* = 2 per group). As shown in Fig. 3, PET imaging clearly depicted the high uptake of [^64^Cu]Cu-DOTA-cetuximab in HCC827 xenografts, which highly overexpress EGFR. Quantification of the PET images revealed that tumor uptakes (SUVmean of [^64^Cu]Cu-DOTA-cetuximab) in HCC827 tumors were exceptionally high (3.17 and 4.41). Although H1650 showed relatively high SUVmean values (1.93 and 2.17), other xenografts of low to moderate EGFR expression showed SUVmean values comparable to those of EGFR-null H520 (range 0.87–1.66, number of mice analyzed: 10/10).

### Comparison of radiolabeled cetuximab tumor uptake with EGFR expression level

**Fig. 4:**
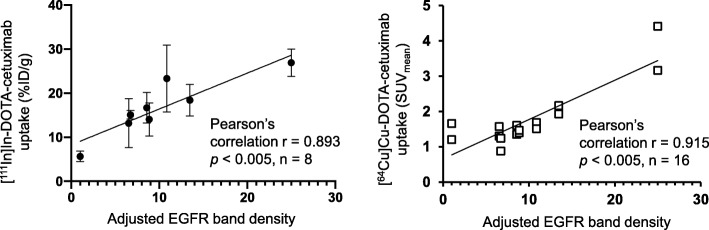
Correlation between epidermal growth factor receptor (EGFR) expression and tumor uptake of radiolabeled cetuximab. (**a**) A significant correlation of EGFR expression level and tumor uptake of [^111^In]In-DOTA-cetuximab was noted within tumor models of non-small cell lung cancer. Each data represents the mean ± SD of n = 5 to 7 per tumor model. (**b**) Tumor uptake of [^64^Cu]Cu-DOTA-cetuximab in positron emission tomography (PET) (mean standardized uptake values [SUV mean]) is correlated with the EGFR expression level. Each data point represents SUVmean of each NSCLC tumor (8 cell lines, *n* = 2 per group) left panel: (**a**), right panel (**b**)

Figure 4 shows the relationship between tumor [^111^In]In-DOTA-cetuximab uptake or [^64^Cu]Cu-DOTA-cetuximab uptake and adjusted EGFR band density. The linear relationships with a positive slope in both graphs indicate the positive correlation between variables. Tumor uptake of [^111^In]In-DOTA-cetuximab uptake strongly correlated with the adjusted EGFR band density (r = 0.893, *p* < 0.005). A strong correlation was also found between tumor uptake of [^64^Cu]Cu-DOTA-cetuximab and adjusted EGFR band density (r = 0.915, p < 0.005). A moderate correlation was observed between [^111^In]In-DOTA-cetuximab tumor uptake and [^64^Cu]Cu-DOTA-cetuximab tumor uptake (r = 0.694).

## Discussion

This study demonstrated the association between NSCLC tumor uptake of radiolabeled cetuximab and EGFR protein expression levels in a xenograft mouse model. Distinguishably high accumulation of radiolabeled cetuximab was noted in EGFR-highly-overexpressing tumors in comparison to the tumors with low and moderate EGFR expression levels, allowing for clear visualization of the tumor in PET imaging. These results suggest the potential usefulness of cetuximab immuno-PET for non-invasive prediction of cetuximab uptake in NSCLC.

The biodistribution study with [^111^In]In-DOTA-cetuximab suggested that radioactivity uptake in the NSCLC xenograft tumors sharply reflects the corresponding EGFR protein expression levels. This result is in line with previous studies of radiolabeled cetuximab employed in colorectal cancer models [12, 17]. The significantly high uptake of [^111^In]In-DOTA-cetuximab in EGFR-highly-overexpressing HCC827 in comparison with xenograft tumors with low to moderate EGFR expression levels suggests the potential of [^64^Cu]Cu-DOTA-cetuximab immuno-PET for detection of EGFR expression status in NSCLC. The large variation in [^111^In]In-DOTA-cetuximab uptake observed within EBC-1 xenografts is perhaps due to the inter-subject heterogeneity of EGFR expression levels. Further investigation such as a side-by-side comparison between [^111^In]In-DOTA-cetuximab distribution and EGFR expression ex vivo might provide some clues; however, it is beyond the scope of the current study. Nevertheless, this inter-subject difference in [^111^In]In-DOTA-cetuximab uptake indicates that radiolabeled cetuximab potentially reflects the inter- or intra-patient heterogeneity of EGFR expression level.

The quantitative analysis of the PET study showed a strong correlation with the adjusted EGFR band density, which is in agreement with the biodistribution study. HCC827 tumors showed exceptionally high SUVmean levels compared to other tumors, which reinforced the potential of [^64^Cu]Cu-DOTA-cetuximab-PET for the identification of NSCLC patients suitable for cetuximab therapy. Meanwhile, there was a small variation in SUVmean values between EGFR low to high expressing tumors. Since various factors may influence tumor uptake of radiolabeled antibodies—such as extravasation from tumor capillaries, diffusion and binding within the tumor interstitium, plasma clearance, internalization, and catabolism in tumor cells [18, 19], and morphological or physical barriers—specific binding to the antigen may not be the dominant factor determining cetuximab uptake level in NSCLC tumor with EGFR expression on its cell surface below a certain level. Indeed, H520 cells form highly vascularized tumors [20]. In addition, because SUV is standardized for body weight, the numerical range of SUV values in mice are not equivalent to those in human. A number of clinical studies have been demonstrated that SUV values for tumor uptake of radiolabeled antibodies vary widely among lesions [9, 21]. Although clinical confirmation is necessary, it is highly likely that [^64^Cu]Cu-DOTA-cetuximab shows SUVmean values of NSCLC tumors in a range sufficient to differentiate cetuximab responsive cases from non-responsive cases.

Another factor limiting the range of SUVmean values would be the low specific activity of [^64^Cu]Cu-DOTA-cetuximab (4.5–30 GBq/μmol). This necessitated high protein dose (100 μg/head) administration in the PET study, which may have competitively inhibited the radiotracer uptake due to receptor saturation in low-EGFR-expressing tumors [22–24]. This difference may also account for the discrepancy in the radiotracer tumor uptake between the biodistiribution study and PET imaging, which showed only a moderate correlation. Tamura et al. [25] reported much higher specific activity of [^64^Cu]Cu-DOTA-trastuzumab (350 GBq/μmol). These results indicate that optimization of the ^64^CuCl_2_ production process is necessary to obtain clinically acceptable specific activity of [^64^Cu]Cu-DOTA-cetuximab in our facility. However, the low specific activity would not affect clinical evaluation of radiotracer uptake because high amount of protein (up to 50 mg) are required to prevent rapid clearance and allow for tumor accumulation of radiolabeled antibodies [21, 26].

Although several large clinical NSCLC trials evaluating the efficacy of chemotherapy plus cetuximab suggested the value of a high EGFR expression level determined by quantitative analysis of IHC or FISH as a predictive biomarker, the robustness of this theory is still controversial [3, 27, 28]. A recently closed, large, randomized phase-3 trial (SWOG S0819) failed to show the clinical benefit of the addition of cetuximab to platinum-based chemotherapy even within the patient subgroup with high EGFR FISH scores [5]. The use of IHC or FISH scores has several concerns such as the definition of positive test results, reproducibility, and the discrepancy between biomarker status determined based on IHC and FISH scores [7], which may have contributed to the subgroup misclassification and led to the controversial results among studies. Unlike histological analysis, immuno-PET can non-invasively reflect dynamic biomarker status at the whole-body level. In case of NSCLC, PET imaging with [^64^Cu]Cu-DOTA-cetuximab by itself could predict the response to therapy as it is indicative of cetuximab tumor accessibility while the therapeutic response are not associated with biomolecular characteristics such as the status of KRAS, PTEN, and EGFR mutations. Therefore, addition of immuno-PET scanning using [^64^Cu]Cu-DOTA-cetuximab to the workup protocol may help increase the accuracy of biomarker status assessment in NSCLC patients. Comparative studies to evaluate the predictive values of IHC and/or FISH scores separately or in combination with immuno-PET based analysis are required to test this hypothesis.

There are several limitations in our study. First, xenografts of established human tumor cell lines may have different characteristics from those of primary human tumors, and thereby EGFR expression levels determined here is not directly comparable to the EGFR-positivity criteria commonly used in clinical setting. Further studies in more clinically relevant models are warranted [4]. In addition, the predictive value of [^64^Cu]Cu-DOTA-cetuximab immuno-PET for cetuximab treatment was not determined. Finally, although suitable main positron energy of ^64^Cu (653 keV) can provide PET images with spatial resolution better than ^86^Y (Emax = 3.1 MeV, with an additional γ-emission of 1.08 MeV (83% abundance)), another positron emitter suitable for antibody imaging [22], there are several concerns regarding the use of ^64^Cu for imaging tumor uptake of antibodies, such as its limited availability and relatively short half-life (12.7 h). Detection of lung legions might be hindered by accumulation of radioactivity in the liver due to the relatively weak in vivo stability of [^64^Cu]Cu-DOTA. But the use of ^64^Cu is a valuable option especially in facilities at which other longer-lived positron emitter isotopes such as ^89^Zr (398 keV, t_1/2_ = 3.3 d) and ^86^Y are not available.

## Conclusion

This study has demonstrated that cetuximab uptake in NSCLC tumors can be assessed by PET using ^64^Cu-labeled cetuximab. Significantly high uptake of [^64^Cu]Cu-DOTA-cetuximab was noted in NSCLC tumors with very high EGFR expression levels compared to tumors with medium or low EGFR expression levels. These results suggest that immuno-PET with [^64^Cu]Cu-DOTA-cetuximab may provide additional information for selection of patients with advanced NSCLC most likely to benefit from cetuximab treatment.

## Data Availability

The datasets used and/or analysed during the current study are available from the corresponding author on reasonable request.

## Abbreviations

DOTA or *p*-SCN-Bn-DOTA: 2-(4-isothiocyanatobenzyl)-1,4,7,10-tetraazacyclododecane-1,4,7,10-tetraacetic acid
EGFR: Epidermal growth factor receptor
IHC: Immunohistochemistry, FISH: Fluorescent in situ Hybridisation
NSCLC: Non-small cell lung cancer
PET: Positron Emission Tomography
ROI: Region of interest
SUV: Standardized uptake value
TKI: Tyrosine kinase inhibitor
